# Intravesical Botulin Toxin-A Injections for Neurogenic Bladder Dysfunction in Children: Summary Update on Last 10 Years of Research

**DOI:** 10.3390/toxins16080339

**Published:** 2024-08-01

**Authors:** Andrea Zulli, Virginia Carletti, Alberto Mantovani, Maria Angela Cerruto, Luca Giacomello

**Affiliations:** 1 Department of Pediatric Urology, Meyer Children’s Hospital IRCCS, 50139 Florence, Italy; andrea.zulli@unifi.it (A.Z.); virginia.carletti@unifi.it (V.C.); alberto.mantovani@meyer.it (A.M.); 2 School of Pediatric Surgery, University of Florence, 50139 Florence, Italy; 3 Department of Surgical Sciences, Dentistry, Gynecology and Pediatrics, University of Verona, 37134 Verona, Italy

**Keywords:** neurogenic bladder, pediatric urology, botulin toxin-A

## Abstract

Neurogenic bladder dysfunction (NB) represents a challenge in pediatric urology. Intravesical botulin toxin-A (BTX-A) bladder injection is part of the armamentarium for the treatment of this condition, usually after failed first-line medical strategies and before the escalation to more invasive options such as neuromodulation or augmented cystoplasty in severe cases. However, there is still a lack of consensus about the appropriate treatment modality for the pediatric population. A review of the last 10 years’ research was performed on the PubMed database by two authors. Articles doubly selected and meeting the inclusion criteria were collected and analyzed for their study type, demographics, neurological disease(s) at diagnosis, BTX-A treatment modality and duration, previous treatment, clinical and urodynamic parameters, adverse events, outcomes, and follow-ups. A total of 285 studies were initially selected, 16 of which matched the inclusion criteria. A cohort of 630 patients was treated with BTX-A at a median age of 9.7 years, 40% of which had a diagnosis of myelomeningocele. The results of the selected publications show the overall efficacy and safety of BTX-A injections in children and confirmed BTX-A as a valuable strategy for NB treatment in pediatric population. Nevertheless, up to now, the literature on this topic offers scarce uniformity among the published series and poor protocol standardization.

## 1. Introduction

Neurogenic bladder dysfunction (NB) is a complex disease which often affects patient life. Beyond treatment burden, these patients often experience significant physical limitations to daily activities and are at risk of social exclusion. In children, the most common cause of NB is reported to be myelomeningocele (MMC). In these patients, daily activities are frequently affected by urinary leakage. Moreover, detrusor overactivity (DO) often results in decreased bladder capacity, low compliance, high pressure, and hydronephrosis. The risk of urinary tract infection (UTI) and renal function deterioration can therefore seriously affect these children’s prognoses and quality of life [1]. Children with NB are classically treated with anticholinergic drugs such as oxybutynin or β-3 agonists and undergo daily clean intermittent catheterization (CIC) to reduce intravesical pressure and protect renal function [2]. However, in 10–15% of patients, these therapies fail due to a refractory overactive bladder or the onset of side effects (such as dry mouth, constipation, and blurred vision). Consequently, the bladder pressure remains high, and urinary symptoms persist. In those resistant patients, intravesical injection of BTX-A is considered an alternative as it can improve symptoms, avoiding surgical interventions such as continent urinary diversion with bladder augmentation [3]. The rationale is that BTX-A is capable of blocking the presynaptic release of acetylcholine from the parasympathetic efferent nerves. The efficacy of this action may result not only from an inhibitory effect on the detrusor muscle but also from some effects which are mediated by alterations in the afferent sensory nerve input. Many studies have demonstrated the efficacy of intravesical BTX-A bladder injection in improving symptoms. However, there is a lack of consensus about the appropriate treatment modality in the pediatric population. This study is a review of select studies published on this topic in the last 10 years, with the final aim being to shed some light on this unclarified yet highly clinically relevant medical problem.

## 2. Material and Methods

A literature review was performed using the PubMed, Cochrane, Ovid-Embase, and Scopus databases and while limiting the research to the last 10 years (January 2013–December 2023). The following keywords were used: “botulinum toxin”, “children”, “adolescent”, “neuropathic bladder”, and “neurogenic bladder”. Articles published in English and involving only patients under 18 years were included in the review. Review articles, case reports, commentaries, editorials, letters, abstracts, and adult series (>18 years) were excluded. Articles describing the use of BTX-A for not-neuropathic bladder dysfunction were also excluded.

**Figure 1:**
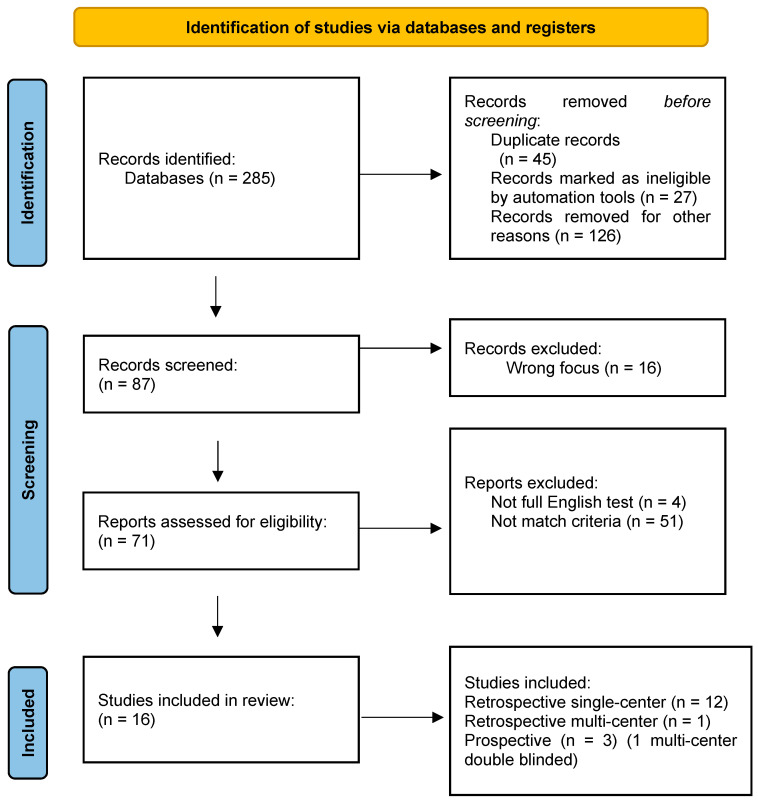
Prisma flow chart: study selection.

All fully published English-language clinical studies on BTX-A were reviewed independently by two authors (V.C. and A.Z.). Only the articles which met the inclusion criteria and were selected by both authors were included (Figure 1). The references of each article were also screened for further research.

For each article, the study type, anagraphic data, neurological disease(s) at diagnosis, BTX-A treatment modality and duration, previous treatment, clinical and urodynamic parameters, adverse events, outcomes, and follow-up data were collected when available.

## 3. Results

A total of 285 studies were collected and screened, and 16 of them matched the inclusion criteria; 12 were retrospective single-center studies, 1 was a retrospective multi-center study, and 3 were prospective, 1 of which was a multicentric, randomized double-blinded study. An overall cohort of 630 patients was reviewed. The median age at the first BTX-A injection was 9.7 years (IQR range: 8.5–11.3). The most common diagnosis was myelomeningocele (250/630, 40%). The other diagnoses were transverse myelitis, lipomeningocele, NB following tumor resection, trauma, sacrococcygeal teratoma, and caudal regression syndrome. Indications of BTX-A treatment varied among the studies and included both clinical and urodynamics parameters. All patients received BTX-A treatment after failed or insufficient conservative treatment with CIC, anticholinergic agents, or a combination of both. The dosage of BTX-A was 10 IU/kg in 8 series [4,5,6,7,8,9,10,11]. Austin et al. performed a comparison among three groups of patients which received 50 IU, 100 IU, and 200 IU, regardless of the patients’ weights [12]. All of the authors diluted the botulinum toxin in normal saline. The number of punctures during the single procedure ranged from 20 to 40. The trigone and bladder neck were excluded as well as the ureteral orifices.

Eight authors performed repeated injections based on symptom recurrence and patients’ satisfaction [5,6,9,10,11,13,14,15]. The time interval between injections was not always reported; when described, it was 13.1 months [5], 11.9 months [6], and 14 months [15]. The reason for re-intervention was a lack of response or detriment of symptoms in all cases. No peri-operative complications were described. The most frequent adverse events were hematuria (5/552, 0.9%) and temporary urinary retention (2/552, 0.4%). Complications were not reported in five papers.

UTIs post BTX-A treatment were reported in 72/552 (13%) patients, even if it was not clear if those were part of the chronic symptoms of the patients or if the UTI rate increased after treatment.

The clinical, demographics, urodynamics parameters, and outcomes are shown in Table 1.

## 4. Discussion

NBs in children, such as those caused by spina bifida and other congenital or acquired conditions, pose significant clinical challenges. These conditions can lead to neurogenic detrusor overactivity (NDO) which, if not managed properly, can result in renal damage and reduced quality of life. BTX-A has emerged as a promising treatment option for managing these dysfunctions. Even with different outcomes, several studies have shown the overall clinical efficacy of BTX-A injections, which are usually adopted as a second-line treatment after anticholinergic drugs and CIC or in combination with either.

As mentioned, the outcomes were reported rather heterogeneously, and it might be difficult to objectively compare the results. First of all, not all of the papers described the post-treatment urodynamic parameters. However, when reported, an increase in bladder capacity and compliance and a reduction in the maximum detrusor pressure were described. Furthermore, two papers reported improvement in preexisting vesicoureteral reflux (VUR).

Early intervention seems to be crucial in managing neurogenic bladder dysfunctions to preserve kidney function. Dik et al. emphasized the importance of initiating BTX-A treatment early in spina bifida patients to prevent renal deterioration. Through this approach, it seems possible to maintain better control of overactive bladders and protect renal health [1].

The British Association of Paediatric Urologists provided comprehensive guidelines for the management of NB in children, including BTX-A as a treatment option [2]. The authors detailed these strategies, highlighting the role of BTX-A in managing bladder dysfunctions when standard therapies fail. The consensus supports BTX-A for its efficacy and relatively minimal invasiveness compared with surgical interventions such as bladder augmentation with urinary diversion.

Figueroa et al., in their single-center experience with BTX-A injections in 17 children, found that dose adjustments and repeated injections significantly improve bladder function and reduce the need for more invasive procedures [3].

Moreover, Sharifiaghdas et al., in their series of 35 children, demonstrated notable improvements in both the post-treatment clinical and radiological parameters, highlighting the therapeutic potential of BTX-A in managing NB [4].

Botulin injections are generally adopted for patients refractory to medical treatment, thus underscoring the need to properly select NB patients who may benefit from the procedure. Danacioglu et al. identified factors predicting the success of BTX-A treatment in children with neurogenic bladders due to myelomeningocele, and they found that some preoperative urodynamic parameters, such as the presence of a low-compliance bladder without DOA potentially predicting therapeutic outcomes, enabling better patient selection and treatment planning [5]. Similarly, Madec et al. discussed the long-term efficacy of repeated BTX-A injections, suggesting that continuous and repeated administration, with a medium interval of 11.9 months, is a sustainable option for managing NB over the long term [6].

The same urodynamic parameters have recently been shown to predict the outcome of relieving bladder outlet obstruction in kidney transplant patients in the adult population, a finding which underlines the relevant clinical value of low-compliance bladders in determining the success of endoscopic procedures in incontinent patients as a whole [16].

Several single-center studies, such as those proposed by Peeraully et al. and Peyronnet et al. [13,15], provide valuable insights into the practical application of BTX-A in pediatric patients. Peeraully et al. reported a decade-long experience with BTX-A injections, noting improvements in bladder function and patients’ quality of life in 71.4% of the patients treated with repeated injections [15]. Peyronnet et al. conducted a multicenter study on children with spina bifida, reinforcing the efficacy of BTX-A treatment in improving bladder compliance and reducing incontinence [13]. In their series, 62.3% of patients were treated with repeated injections, with an overall clinical success rate of 66%. Additionally, Sekerci et al. [14] reported the outcomes of up to five repeated BTX-A injections in children with refractory NDO, demonstrating sustained efficacy and manageable safety profiles over multiple treatment cycles.

As shown, different treatment modalities exist worldwide, and the need for a summary was already detected by Wu et al. in 2021 [20]. In his review on botulin injections in children, 16 articles were selected, and all but one reported improvement in clinical parameters such as incontinence, VUR, UTIs, and hydronephrosis. Moreover, although the urodynamics parameters considered in the included studies were various, a decrease in detrusor pressure and improvement in bladder capacity and compliance were described.

After the publication of this review, four more articles were published, all of which are retrospective single-center studies [4,5,6,21]. Therefore, the current literature still lacks prospective trials on BTX-A treatment, since most of the studies are retrospective.

Three prospective studies were included in this review [7,8,12]. Hui et al. conducted a prospective multicentric trial, investigating the safety and efficacy of trigonal BTX-A injections for children with NDO secondary to spinal cord injuries. BTX-A injections are usually performed along the bladder’s mucosal surface, avoiding the trigonal and bladder neck area. Despite this, the authors treated 33 patients with trigonal injections, noting a reduction in urinary incontinence episodes, increased voiding volumes, and improvement in the Incontinence Quality of Life questionnaire in all patients. Their results confirmed that this approach is both safe and effective, providing substantial symptom relief without significant adverse effects [7].

In a prospective multicentric, randomized double-blind trial by Austin et al., the population was divided into three groups according to the BTX-A dose injected. The first group (group 50 U) received 50–72 IU; the second group (group 100 U) received 96–144 IU; and the third group (group 200 U) received 168–200 IU. The authors observed a dose-dependent increase in functional bladder capacity which was statistically significant for the 200 U versus 50 U doses (*p* = 0.0055). A significant improvement from the baseline in storage pressures was also seen in the 200 U arm when compared with the 50 U group (*p* = 0.0157). There was an increase in the maximum cystometric capacity in all dosage groups, even if there were no statistically significant differences. The duration of the BTX-A effect, based on the median time for patients to require retreatment, did not differ significantly between groups. Reductions in UI episodes were similar across doses. The authors are also conducting a long-term extension study to evaluate the continued safety and efficacy following repeated treatments [12].

Mohajerzadeh et al., in their prospective trial, described a significant reduction in post-void residual volume and an increased cystometric bladder capacity after the injections. However, the study failed to demonstrate any significant improvement in the flow average and peak flow time [21].

Similar conclusions were found by Marte et al. [19]. The authors, in this retrospective single-center study, observed a positive effect on dryness and quality of life, with 38/47 patients achieving dryness with CIC while 9/47 patients improved their incontinence but still needed pads.

Tarcan et al. also reported important improvements, with 30/31 patients being dry with CIC, a 53% reduction in the maximum detrusor pressure, and a 51.5% increase in maximum cystometric capacity 6 weeks after the injections. Moreover, a 324% increase in mean bladder compliance and a 57% increase in mean intermittent catheterization volumes was found [18].

This review suffers from different biases. The main limitations are certainly the small number of studies which matched the inclusion criteria, the retrospective nature of many studies included, and the difficulties in perfectly matching the results of each cohort.

A larger number of high level of evidence data is needed in the future in order to assess the results induced by BTX-A treatment in children. In particular, a longer follow-up period is advised to evaluate the long-term effects of BTX-A injections on the bladder wall.

## 5. Conclusions

BTX-A represents a significant advancement in the management of neurological bladder dysfunctions in children. It offers a mini-invasive treatment which can improve bladder function, protect renal health, and enhance quality of life. The last 10 years of research have confirmed its efficacy and safety, providing prospective studies to reinforce the level of evidence on this topic. Patients must be counseled for possible repeated injections to maintain the clinical results. For this reason, the treatment itself is strictly tailored to patients’ responses, and successful outcomes depend on appropriate patient selection, careful dose management, and consideration of long-term treatment strategies. Some questions remain unanswered, such as the overall length of multiple cycles of injections and the differences between the outcomes of injection alone versus BTX-A combined with other medications. Studies have investigated different outcomes which do not appear to be uniform and with unstandardized BTX-A protocols, with inevitable bias in comparing the results. Further studies with larger sample sizes and adequate control groups should be conducted to confirm these observations.

Continued research and clinical experience will further refine BTX-A’s use and optimize the outcomes for pediatric patients with neurogenic bladder disorders.

## Figures and Tables

**Table 1:** Clinical, demographics, and urodynamics parameters and outcomes.

Reference	Year	Type of Study	Number of Patients (N)	Mean Age (Years)	Diagnosis (N)	Inclusion Criteria	Treatment Modality	Preoperatory Urodynamic Parameters	Previous Treatments	Treatment Duration	Follow-Up	Adverse Events (N)	Outcomes
[4]	2022	Retrospective, single center	35	9.5	Overactive neurogenic bladder (not specified)	Patients aged 1–18 years with the diagnosis of overactive neurogenic bladder proven by clinical evaluation, nervous system imaging, and urodynamic studies and with overreactive bladder symptoms and deterioration of urinary tract function and imaging	10 U/kg diluted with 6 mL of normal saline; 20–30 points of injection in the bladder intra detrusor and away from the bladder neck and ureteral orifices	Frequency, nocturia, urge urinary incontinence (UUI), urgency, enuresis, and hydronephrosis	CIC	Single injection	6 months	Positive urine culture without symptoms (3); UTI (1); temporary urinary retention (1)	93% satisfied
[16]	2021	Retrospective, single center	34	15.5	Myelomeningocele (14), transverse myelitis (3), lipomeningocele (7), and other (10)	Detrusor overactivity refractory to medical therapy, intolerance of oral therapy (oxybutynin, tolterodine, trospium, glycopyrrolate, solifenacin, or mirabegron, either individually or concomitantly), urinary incontinence between CIC and hydronephrosis refractory to medical therapy, symptomatic DO or DO on UDS (defined as a rise in detrusor pressure >15 cm H_2_O above baseline) or low compliance (reflected in UDS as a substantial rise in pressure at an appropriate age-specific volume to maintain CIC at 4 hourly intervals), or presence or worsening of hydronephrosis	4.3 U/kg, 20 points of injection in the bladder, and trigone-sparing sites (1 mL per site)	High- (Pdetmax > 20 cm H_2_O) and low-pressure bladder (Pdetmax ≤ 20 cm H_2_O), high compliance (>10 mL/cm H_2_O), low compliance (≤10 mL/cm H_2_O), and finally low (<50% of expected cystometric capacity (CC)) and normal bladder capacity (≥50% of expected CC), defined as observed or estimated bladder capacity (mL/mL)	Oxybutynin, tolterodine, trospium, glycopyrrolate, solifenacin, or mirabegron, either individually or concomitantly	Single injection	NR *	NR	Success 13 (38.2%), failure 21 (61.8%)
[5]	2021	Retrospective, single center	62	9.1	Myelomeningocele (62)	CIC use before BoNT-A injections and failed or non-tolerated oral anticholinergic treatments	10 U/kg diluted with 0.9% NaCl (maximum dose of 300 U); 20–30 points of injection	27.4% (n = 17) of patients had hypocompliant bladders without detrusor overactivity (DOA), 38.7% (n = 24) of patients had normal bladder compliance with DOA, and 33.9% (n = 21) of patients had hypocompliant bladders with DOA	Anticholinergic agents + CIC	Single injection. After mean follow-up of 28.5 12.2 months (range: 11–72 months), 54.8% (n = 34) of patients had repeated injections (from two to seven injections), resulting in total number of 117 BoNT-A injections performed. Median interval between two injections was 13.1 months.	28.5 months	Febrile urinary infection (4)	Success was achieved in 64.5% (n = 40) of patients after first injection. The mean MCC increased from 172.4 (45.6 mL) to 236.3 (67.2 mL). The mean bladder compliance increased from 14.8 (8.1 mL/cm H_2_O) to (19.3 7.4 mL/cm H_2_O), and the mean maximum detrusor pressure decreased from 56.7 (18.8 cm H_2_O) to 36.6 (10.1 cm H_2_O). Reflux disappeared in 53.8% (n = 14) of the ureters, improved in 26.9% (n = 7), and remained unchanged in 19.2% (n = 5).
[6]	2021	Retrospective, single center	17	8	Open dysraphism (1); closed dysraphism (7); post-tumoral resection (3); post-traumatic (1); post-encephalo-myelitis (3); Hinman syndrome (2)	Patients referred for neurogenic bladder-related issues and treated with at least 4 IDI-TBA	10 U/kg (maximum of 300 U) diluted in normal saline; 30 points of injection in detrusor (1 mL/spot)	Bladder capacity (mean) = 112 mL; bladder capacity ratio (%) = 36.1; detrusor pressure (mean) = 42 cm H_2_O	Anticholinergic agents + CIC	A total of 95 IDI-TBA, median per patient of 5 [4,5,6,7,8] (8: n = 2—11.8%, 7: n = 4—23.5%, 6: n = 0; 5: n = 7—41.2%, 4: n = 4—23.5%); median interval = 357 days in case of clinical or cystometric deterioration	57 months	Pielonefritis (14 in 6 patients); changes in renal morphology (3)	Bladder capacity (mean): 200 mL after 1st treatment; 220 mL at the end of treatment. Bladder capacity ratio (%): 80.3 after 1st treatment; 57.1 at the end of treatment. Detrusor pressure (mean): 8 cm H_2_O after 1st treatment; 16 cm H_2_O at the end of treatment.
[7]	2020	Prospective, multi-centric	33	15.6	Traumatic injury 28; spinal surgery 5	Spinal cord injury with stable neurologic status; urodynamic detrusor overactivity; resistance or noncompliance to two or more anticholinergic medications; participants’ parents or their caregivers agreed to perform clean intermittent catheterization	10 U/kg (maximal dose = 200 U) diluted in a total of 30 mL sterile saline; 30 points of injection	Maximum detrusor pressure = 45.83 cm H_2_O (mean); volume = 163.44 mL (mean)	Anticholinergic agents + CIC	Single injection	12 weeks	Mild transient hematuria during first week after injection (3)	Maximum detrusor pressure = 32.15 cm H_2_O (mean); volume = 246.01 mL (mean). Urinary incontinence episodes (mean): 4.01 (prior), 2.76 (post). Voiding volume (mL): 183.16 (prior), 280.02 (post). Dryness: n = 0 (prior), 4 (post). I-QoL = incontinence quality of life questionnaire: 40.68 (prior), 50.13 (post); *p* < 0.05 for all outcomes.
[12]	2020	Prosepctive, multi-centric, randomized, double-blind	113	11.3	Spinal dysraphism (99); spinal cord injury (13); transverse myelitis (1)	Patients inadequately managed with anticholinergic agents, regularly using CIC (≥3 times/day for ≥3 months before screening), having ≥ 4 episodes of daytime UI over a 2 day diary	20 intradetrusor injections of 0.5 mL excluding the trigone; Group 50 received 50 U, Group 100 received 100 U, Group 200 received 200 U	Group 50: maximum detrusor pressure during storage phase of 58.2 cm H_2_O (mean); maximum cystometric capacity of 169.1 mL (mean); involuntary detrusor contraction = 94.4%. Group 100: maximum detrusor pressure during storage phase of 56.5 cm H_2_O (mean); maximum cystometric capacity of 179.2 mL (mean); involuntary detrusor contraction = 88.1%. Group 200: maximum detrusor pressure during storage phase of 56.7 cm H_2_O (mean); maximum cystometric capacity of 202.3 mL (mean); involuntary detrusor contraction = 92.6%	Anticholinergic agents + CIC	Single injection	48 weeks	UTI (33)	Improvements from baseline in number of daytime UI episodes in all dose groups; after 6 weeks, the majority of patients in each group reported “great improvement” or “improvement” in TBS; dose-dependent increase in functional bladder capacity, measured by volume at first morning catheterization recordings; significant improvement from baseline in urodynamic storage pressures; and increase from baseline to week 6 in maximum cystometric pressure in all dose groups
[8]	2020	Retrospective, single center	26	7.7	Mielomeningocele (26)	Children with MMC older than 3 years of age who had moderate-to-severe urinary incontinence with urodynamically proven neurogenic detrusor over activity (NDO). Spontaneous detrusor contraction during filling phase causing detrusor pressure increase to >15 cm H_2_O from the baseline; high detrusor pressure (>40 cm H_2_O)	10 U/kg diluted in 20 mL of normal saline; at least 40 points of injection sparing trigone and ureteral orifices	Mean maximal detrusor pressure (cm H_2_O), maximal cystometric capacity (mL), mean detrusor compliance (mL/cm H_2_O), median urinary incontinence score (0–3), and urinary incontinence score (0–3)	Anticholinergic agents + CIC	Single injection	1 year	NR	8 of 12 (66.6%) patients became completely dry between 2 consecutive clean intermittent catheterizations, which were maintained in 6 of 12 (50%) patients
[17]	2020	Prospective, mono-centric	20	7.7	Myelomeningocele: 18; post-surgery: 2	Patients who did not respond to anticholinergic medications or could not tolerate side effects	2 mg/kg per day diluted in normal saline; 40 square-shaped areas 1 cm on each side away from trigone; approximately 10 U were injected into posterior and anterior walls	Mean: Flow rate in second two (mL/s) = 6.6; flow time of diuresis (s) = 59.77; peak flow time (s) = 16.98; flow average = 7.71; discharged volume (mL) = 106.84; maximum detrusor muscle filling pressure (cm H_2_O) = 86.03; maximum flow (mL) = 13.87; acceleration (mL/s^2^) = 1.25; post-void residual volume (mL) = 9.02; compliance (mL/cm H_2_O) = 7.85; cystometric bladder capacity (mL) = 115.41	Oxibutinine, anticholinergics	Single injection	NR	NR	3 months later (mean): flow rate in second two (mL/s) = 7.09, *p* = 0.60; flow time of diuresis (s) = 48.61, *p* = 0.03; peak flow time (s) = 15.49, *p* = 0.12; flow average = 7.30, *p =* 0.32; discharged volume (mL) = 128.90, *p* < 0.005; maximum detrusor muscle filling pressure (cm H_2_O) = 72.03, *p* < 0.005, maximum flow (mL) = 13.57, *p* = 0.30 acceleration (mL/s^2^) = 1.28, *p* = 0.34; post-void residual volume (mL) = 5.47, *p* = 0.02; compliance (mL/cm H_2_O) = 2.12, *p =* 0.002; cystometric bladder capacity (mL) = 134.38, *p* = 0.002
[15]	2019	Retrospective, single center	28	11.1	Open myelomeningoceles (17), tethered cord within lipomatous mass with associated sacral agenesis (1), cerebral palsy and idiopathic congenital dystonia (10)	Involuntary voiding or urinary incontinence between CICs, where all received oral anticholinergics and had urodynamically proven impaired bladder compliance, DO, or reduced bladder capacity	375 U or 500 U (not in those <12 years) of DysportTM diluted with normal saline to total volume of 20 mL (concentration of either 18.75 U/mL or 25 U/mL); 20 points of injection equally distributed in detrusor muscle, sparing trigone	NR	Anticholinergic agents + CIC	Repeated injection if no effects or waning response; mean = 14 months	25 months	Difficulty initiating voiding (1); UTI (1)	8 did not improve, 14 mildly improved, 6 dry
[13]	2018	Retrospective, multicenter	53	8.5	Myelomeningoceles (25), closed spinal dysraphisms (28)	Patients under CIC and not receiving therapeutic botulinum toxin (for any indication) in previous 3 months, with no history of myasthenia or coagulation disorders	No standardized dose due to retrospective nature of study	14 patients (26.4%) had poor compliance in bladder and detrusor overactivity, 11 patients (20.7%) had poor compliance in bladder without detrusor overactivity, and 18 patients (34%) had detrusor overactivity with normal bladder compliance. In 10 patients, pre-injection urodynamic data were not available or incomplete.	Anticholinergic agents + CIC	33 (62.3%) had repeated injections (from 2 to 8 injections) resulting in total number of 141 IBTX-A runs performed. Time interval between injections not reported.	3.7 years	Urinary tract infections (3)	CLINICAL: Most patients were clinically improved after first IDBTX-A run. The clinical success rate was 66%. URODYNAMIC: Compliance (mL/cm H_2_O) = 9.9 (prior), 16.3 (post) (mean); *p* < 0.05. Maximum cystomanometric capacity (mL) = 184.4 (prior), 268.8 (post) (mean); *p* > 0.05. Maximum detrusor pressure (cm H_2_O) = 47.3 (prior), 34.5 (post) (mean); *p* < 0.05.
[14]	2017	Retrospective, single center	19	10.3	Myelodysplasia (19)	NDO due to myelodysplasia refractive to standard treatment protocol with anticholinergics and CIC	100 U diluted with 10 mL of normal saline; 20 points of injection into bladder, sparing trigone; each injection had volume of 1 mL and held 10 U	Maximum cystometric capacity, maximum detrusor pressure, compliance	Anticholinergic agents + CIC	1–5 injections (every 3 months)	4 years	Hematuria (2)	Significant improvements in mentinal parameters after repeat injections
[9]	2015	Retrospective, single center	53	8	Spina bifida (18); acquired spinal cord injury (4); cerebral palsy (3); trnasverse myelitis (1); intraspinal lipoma (1); post pelvic surgery (1); acquired brain injury (1); idiopathic (24)	Failure of conservative treatment	10 U/kg diluted in normal saline (maximum 300 U). Multiple injections were distributed throughout detrusor at 10 units/kg to maximum of 300 units. For intrasphincteric injections = 3 U/kg diluted in normal saline (maximum of 100 U)	NR	Anticholinergic agents, CIC, tamsulosin, midazolam	134 injections in 53 children (106 intravesical, 23 intrasphincteric, 5 combined); mean of 2.57 injections per patient (range: 1–11); time interval not reported	26–79 months	UTI in 2 week period post injection (13)	After each Botox injection, all children responded to meet ICCS category of response; >90% reduction in symptoms
[10]	2015	Retrospective, single center	22	10	Myelomeningocele (10); MAR (3); spinal cord trauma (3); tethered cord (2); caudal regression syndrome (2); sacrococcygeal teratoma (1); transverse myelitis (1)	Failure of CIC and anticholinergic treatment	10 U/kg diluted with normal saline (maximum 300 U) injected into detrusor along posterior and lateral walls while sparing trigone	Cystometric bladder capacity (mean) = 227 mL; mean maximum detrusor (mean) = 63 cm H_2_O; compliance (mean) = 4.3 mL/cm H_2_O	Anticholinergic agents + CIC	Four patients in cohort (18%) had received two or more BTIs; time interval not reported	NR	NR	Cystometric bladder capacity improved by 46% (227 vs. 331 mL, *p* = 0.008). The mean maximum detrusor pressure decreased by 43% (63 vs. 44 cm H_2_O, P Z 0.002), and mean compliance improved by 104% (4.3 vs. 8.8 mL/cm H_2_O, *p* = 0.001), with urodynamics performed at 12 weeks following procedures. Overall, 54% (n = 12) had improved continence after initial BTI, whereas 45% (n = 10) achieved complete continence with prescribed CIC. A total of 75% of AI patients (n = 3/4) were continent with CIC after initial BTI. The mean duration of clinical improvement after the initial BTI was 4.6 months (range: 0–18).
[11]	2014	Retrospective, single center	37	7.5	Spina bifida (29); syrinx (1); cerebral palsy (4); Guillain–Barre syndrome (1); spinal cord hemangioma (1); post-meningitis sequelae (1)	Neurogenic detrusor overactivity refractive to high-dose anticholinergics	10 IU/kg (maximal 300 IU), diluted in normal saline to concentration of 10 U/cc	NR	Anticholinergic agents + CIC	Repeated injections (4 patients received 2 or more injections for recurrence of symptoms); time interval not reported	11 months	NR	Patients with anticholinergics intolerance seen to be more effective after BTX-A injection than those with anticholinergic refractory
[18]	2014	Prospective, single center	31	9.7	Myelomeningocele	NB with urinary incontinence managed without success with CIC and oxybutynin for at least 2 months	10 IU/Kg (maximum = 300 IU), diluted in normal saline (1 mL in each injection, 20 or 30 injections)	P = Pdetmax (cm H_2_O); MCC = maximum cystometric capacity (mL); BC = bladder compliance (mL/cm H_2_O)	CIC and oxybutynin for at least 2 months	One or more injections, depending on follow-up	29 w	9 UTI	30 with dryness with CIC, P reduction of 53%, MCC reduction of 51.5%, 324% increase in BC
[19]	2013	Retrospective, single center	47	10.7	Myelomeningocele	Neurological patients with overactive or poorly compliant bladders on CIC and resistant or non-compliant to pharmacological therapy; inceontinence with CIC	12 IU/Kg (maximum = 200 IU), diluted in 20 cc of normal saline	Mean leak point volume before and after injection (124.8 mL vs. 207 mL), mean leak point pressure before and after injection (38.2 vs. 38.4), specific capacity at 20 cm H_2_O (69.8 vs. 152.6)	Anticholinergic agents + CIC	One or more injections, depending on control, at 12 weeks or after recurrence of symptoms (6–9 m)	5.7 y	38 with slight hematuria for 2–3 days, 2 with UTIs, 2 with gastric pain, 2 with facial flushing, 5 with mild hypostenia	37 with dryness with CIC (10 with Ach therapy), 9 with improved incontinence

* NR = not reported.

## Data Availability

Not applicable.
